# The Effect of Transition to Home Care Model on the Outcomes of Premature Infants and Their Parents: A Systematic Review

**DOI:** 10.3390/children13070876

**Published:** 2026-06-30

**Authors:** Lia Kamila, Guswan Wiwaha, Ari Indra Susanti, Aly Diana, Dany Hilmanto

**Affiliations:** 1 Doctoral Study Program in Medical Science, Faculty of Medicine, Universitas Padjadjaran, Sumedang 45363, Indonesia; 2 Faculty of Midwifery, Institut Kesehatan Rajawali, Bandung 40184, Indonesia; 3 Public Health Department, Faculty of Medicine, Universitas Padjadjaran, Sumedang 45363, Indonesia; ari.indra@unpad.ac.id (A.I.S.); aly@unpad.ac.id (A.D.); 4 Pediatric Department, Faculty of Medicine, Universitas Padjadjaran, Sumedang 45363, Indonesia; dany.hilmanto@unpad.ac.id

**Keywords:** premature infant, parents, transitional care, post-discharge follow-up, neonatal intensive care unit, discharge planning, continuity of care, family-centered care, care coordination, systematic review

## Abstract

**Background:** Preterm infants remain vulnerable after discharge from the neonatal intensive care unit (NICU), yet the key components of effective hospital-to-home transitional care remain unclear. **Objectives:** This systematic review synthesized evidence on hospital-to-home transitional care models for preterm infants and identified intervention characteristics associated with post-discharge infant and parental outcomes. **Methods:** This PRISMA 2020-guided systematic review was registered in PROSPERO (CRD420251011887). The protocol was amended to remove the planned meta-analysis because of substantial heterogeneity. Searches were conducted in EBSCO-MEDLINE, Scopus, PubMed, and the Cochrane Library in March 2025 and updated in March 2026. Quantitative and mixed-method studies evaluating hospital-to-home transitional care for preterm infants were included. Findings were synthesized narratively using a SWiM-based approach. **Results:** Twenty-one studies were included, comprising 4142 study units across infants, parents/caregivers, or infant–parent dyads. Interventions were initiated during NICU admission, at discharge, or after discharge and commonly included discharge preparation, parental education, telehealth support, and care coordination. Several studies reported favorable outcomes related to infant growth, feeding, length of stay, readmission, healthcare utilization, and parental readiness or caregiving competence, although findings varied across intervention types, study designs, and outcome measures. Three non-comparative studies provided additional evidence on feasibility and acceptability. **Conclusions:** Hospital-to-home transitional care may benefit preterm infants and their families when delivered as a multicomponent, family-centered pathway extending beyond discharge. However, the evidence remains heterogeneous, and practice implications should be interpreted cautiously. Larger comparative studies using standardized infant-centered outcomes are needed, particularly in low- and middle-income settings.

## 1. Introduction

Preterm birth (<37 weeks gestation) remains a leading cause of neonatal mortality and long-term morbidity globally, with an estimated 13.4 million babies born preterm in 2020 and a disproportionately higher burden in low- and middle-income countries (LMICs) [1,2]. Due to physiological immaturity, preterm infants face high risks of severe complications, prolonged hospitalization, and adverse early-life outcomes that often persist beyond discharge [3,4,5,6,7].

The transition from NICU to home is a critical period in the continuum of care. Even after discharge, preterm infants remain clinically vulnerable, requiring ongoing monitoring and nutritional support [8,9]. Although transition-to-home care may also be relevant for other neonatal populations, including infants receiving palliative or perinatal hospice care for life-limiting conditions, the present review focuses specifically on preterm infants because of their ongoing clinical vulnerability, feeding needs, growth concerns, and risk of post-discharge healthcare utilization [10]. Suboptimal post-discharge outcomes, such as poor growth and readmission, frequently result from fragmented continuity of care [7] and parental unpreparedness. Many parents feel ill-equipped to manage their infant’s complex needs due to limited hands-on training and poor coordination between hospital and community services [11,12,13,14,15,16]. Thus, improving infant outcomes requires both strengthening parental preparedness and ensuring seamless care continuity across settings.

Hospital-to-home transitional care models have been developed to address these challenges, typically combining structured discharge preparation, caregiver education, psychosocial support, and coordinated follow-up [17,18]. While several studies suggest these interventions can improve infant growth, breastfeeding success, and healthcare utilization [18,19,20,21,22], the evidence remains inconsistent. Substantial heterogeneity in intervention components, timing, duration, and delivery methods makes it difficult for healthcare providers to determine which specific strategies are most effective and feasible to prioritize [23,24,25].

While several systematic reviews have explored discharge planning or early discharge programs for preterm infants [19,20,23,26,27], significant gaps remain that limit their applicability to current clinical practice. Previous reviews have predominantly focused on isolated intervention components—such as Family Integrated Care (FiCare) alone [28,29], early discharge protocols without post-discharge follow-up, or parental education exclusively [30,31,32]. Furthermore, many existing reviews conceptualize discharge as a single event rather than a continuous transition process, thereby failing to evaluate the integrated pathway from NICU to home and community care. There is also a lack of recent syntheses that explicitly examine how parental outcomes (e.g., readiness and confidence) may function as potential explanatory pathways.

To address these limitations, this systematic review aims to synthesize and critically analyze the available evidence on hospital-to-home transitional care models by evaluating them through a continuum-of-care framework. Unlike previous reviews, this study seeks to identify multicomponent intervention characteristics—including timing, duration, and service linkage—that may be associated with improved post-discharge infant health outcomes, while also exploring the potential role of parental preparedness in supporting infant well-being.

## 2. Materials and Methods

### 2.1. Design

The Preferred Reporting Items for Systematic Reviews and Meta-Analyses (PRISMA) 2020 statement [33,34] was used as guidance for this systematic review. The review protocol was registered in PROSPERO (ID: CRD420251011887). The PRISMA checklist is available in the Supplementary File S1. The protocol was subsequently amended in PROSPERO to remove the planned meta-analysis because the included studies were not sufficiently homogeneous to support quantitative synthesis.

### 2.2. Sources of Information and Search Strategy

The initial literature search was conducted in March 2025. The protocol was subsequently amended in PROSPERO to remove the planned meta-analysis, as the included studies were not sufficiently homogeneous in study design, intervention characteristics, and outcome reporting to support a valid quantitative synthesis. The search was updated in March 2026 to capture the most recent evidence, and newly identified studies were included if they met the prespecified eligibility criteria. Four electronic databases were searched: EBSCO-MEDLINE, Scopus, PubMed, and the Cochrane Library. The four databases were selected because they provide broad coverage of biomedical, neonatal, and evidence-based healthcare literature relevant to transitional care for preterm infants, and the search was supplemented by manual screening of reference lists to enhance comprehensiveness. The search strategy used database-specific syntax and free-text terms related to preterm infants, neonatal intensive care units, and transitional care. An experienced medical librarian assisted in developing the search strategy. No publication-year restrictions were applied. However, only studies published in English were included during the screening process because resources for translation of non-English articles were not available. Reference lists of included studies were also screened manually. Grey literature was not included, as the search was restricted to peer-reviewed databases to maintain methodological consistency. In this review, grey literature refers to sources such as theses or dissertations, conference abstracts or proceedings, institutional reports, policy reports, preprints, and unpublished manuscripts. Complete database-specific search strategies, including search dates, exact search strings, field tags or database-specific syntax where applicable, filters or limits applied, and the number of records retrieved from each database, are provided in Supplementary File S2, in accordance with PRISMA 2020 Item 7. No separate controlled vocabulary terms, such as MeSH terms, were manually combined with the free-text strategy; PubMed Automatic Term Mapping was allowed where applicable.

The review question and eligibility criteria were structured using the PICOS framework (Table 1), which guided the selection of studies.

Table 2 presents the search strategies used for each database and the number of records retrieved.

### 2.3. Selection Criteria

The eligibility criteria were intentionally defined to balance sensitivity and specificity. This ensures the inclusion of relevant transitional care interventions while maintaining conceptual clarity. The study selection process was conducted in two stages. First, titles and abstracts were independently screened by two reviewers (LK and AIS) against the predefined eligibility criteria. Records judged as potentially relevant by either reviewer were retrieved for full-text assessment. Second, the full texts of potentially eligible articles were independently assessed by the same two reviewers (LK and AIS). Disagreements at both the title/abstract and full-text screening stages were resolved through discussion, and when consensus could not be reached, a third reviewer (GW) was consulted. No automation tools were used to make eligibility decisions. The inclusion and exclusion criteria applied in this review are presented in detail in Table 3.

### 2.4. Data Extraction

The data were independently extracted by two reviewers (LK and AIS). The data were extracted from the included studies using a standardized and pilot-tested data extraction form. The extracted information included study characteristics (author, year, country, and study design), participant characteristics, intervention details (type, components, provider), comparator (if applicable), outcomes, intervention timing, duration of intervention, follow-up duration, and key findings.

The accuracy and completeness of the extracted data were verified by the third reviewer (GW). Discussions with additional reviewers (AD and DH) were conducted to resolve any discrepancies until consensus was reached. For the reported missing and incomplete data, attempts were made to contact the study’s authors. Otherwise, the analysis was based on the available data. When studies reported multiple follow-up time points, data from the final follow-up were extracted to ensure comparability across studies. The extracted data are presented in Supplementary File S3.

### 2.5. Risk-of-Bias Assessment

The methodological quality of the included studies was independently assessed by two reviewers (LK and AIS), with verification by additional reviewers (GW and AD). Any disagreements were resolved through discussion until consensus was reached.

Different risk-of-bias assessment tools were applied according to the methodological characteristics of each study because of the heterogeneity of study designs. The Cochrane Risk-of-Bias 2 (RoB 2) tool [35] was used to assess Randomized Controlled Trials (RCTs). This tool evaluates domains including the randomization process, deviations from intended interventions, missing outcome data, measurement of outcomes, and selection of reported results. The Risk of Bias in Non-randomized Studies of Interventions (ROBINS-I) tool was used to assess non-randomized studies, including quasi-experimental and observational designs [36]. This evaluates bias due to confounding, participant selection, classification of interventions, deviations from intended interventions, missing data, outcome measurement, and selective reporting. Studies that do not have a comparator group were not formally assessed with ROBINS-I. This is because this tool is intended for comparative non-randomized studies. Instead, these studies were described narratively in terms of their design, intervention characteristics, and reported outcomes.

### 2.6. Data Analysis

Meta-analysis was not performed because the included studies exhibited substantial clinical and methodological heterogeneity. This heterogeneity arose from variations in intervention components, timing, delivery modes, study designs, outcome definitions, measurement instruments, follow-up durations, and participant characteristics. Although outcome-specific quantitative synthesis was considered, the limited number of studies available for each outcome and the lack of sufficient comparability across studies made statistical pooling inappropriate. Accordingly, the findings were synthesized narratively in accordance with the Synthesis Without Meta-analysis (SWiM) reporting guidelines [37]. Following the SWiM framework, studies were grouped based on intervention characteristics:

- Timing of initiation (pre-discharge, at discharge, post-discharge)
- Format (single-component versus multicomponent)
- Delivery modality (in-person, telehealth/app-supported, or combined)
- Primary outcome focus (infant-centered versus parent-centered).

To provide a structured synthesis, the direction of the effect (e.g., favorable, unfavorable, or no effect) for each outcome domain was tabulated across studies. Standardized metrics were not calculated due to heterogeneity; instead, the synthesis prioritizes the consistency of effects across studies and the risk of bias of the contributing evidence. Patterns and consistencies across studies were identified, with particular attention to patterns in the direction and consistency of findings across intervention components and outcome domains. To facilitate structured interpretation of the findings, outcome domains were summarized narratively according to intervention characteristics, direction of effect, consistency of findings, and methodological considerations. Where mixed-methods studies were included, only quantitative findings contributed to the narrative synthesis of effectiveness outcomes. Qualitative findings were not formally synthesized.

## 3. Results

**Figure 1:**
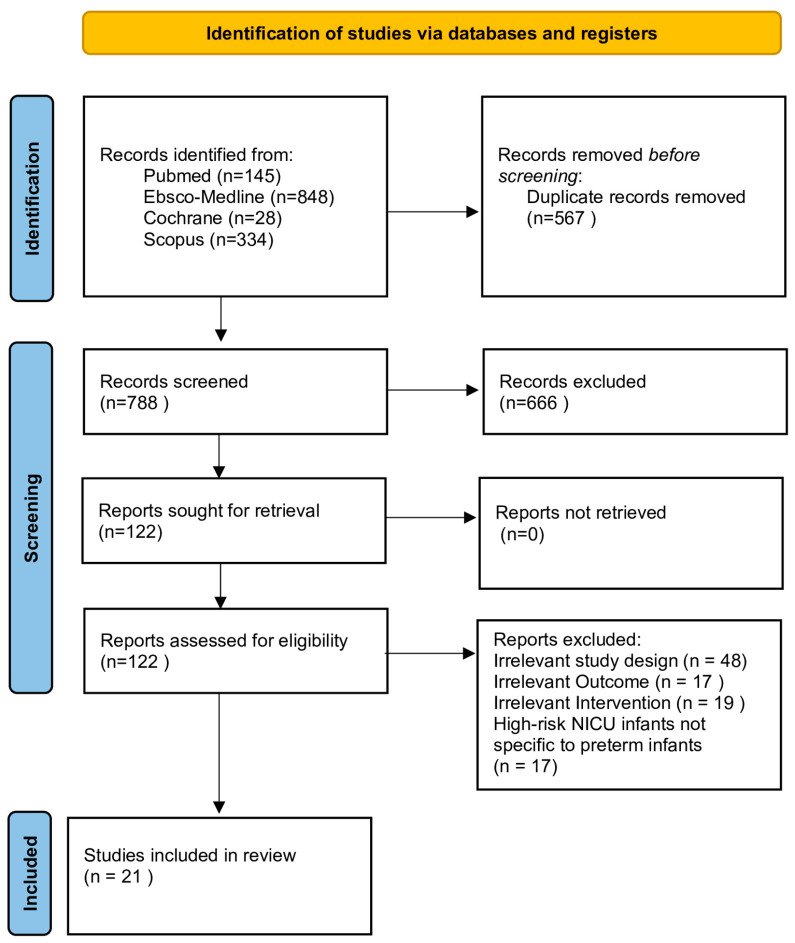
PRISMA flow.

A total of 1355 records were identified through database searching. After removing 567 duplicates, 788 records remained for screening. Following title and abstract screening, 666 records were excluded. A total of 122 full-text articles were assessed for eligibility, all of which were successfully retrieved. Of these, 101 articles were excluded for not meeting the inclusion criteria. The list of excluded full-text studies and the reasons for exclusion are provided in Supplementary File S5. Finally, 21 studies were included in the review. The study selection process is presented in Figure 1.

### 3.1. Characteristics of Included Studies

In this review, 21 studies published between 1999 and 2026 were included. Across these studies, the total reported sample was 4142 study units, including preterm infants, parents/caregivers, or infant–parent dyads depending on the design and reporting of each primary study. Therefore, this number should not be interpreted as a uniform count of individual infants or parents across all studies. The included studies were conducted across a range of countries and healthcare settings, including North America, Europe, Asia, and Africa. These contexts included the United States, China, the Netherlands, Denmark, the United Kingdom, Spain, Sweden, Iran, Thailand, Turkey, Kenya, and Israel. This geographic diversity provides useful contextual insight, although most studies were conducted in high-income or upper-middle-income settings.

Reported sample sizes ranged from 26 to 686 study units across the included studies. Also, the included studies comprised a range of methodological approaches, including experimental, quasi-experimental, observational, and mixed-methods designs. Nine of 21 studies (43%) were randomized controlled trials [29,30,31,32,38,39,40,41,42], and another nine (43%) used quasi-experimental designs [18,19,27,43,44,45,46,47,48]. Observational studies were represented by 1 cohort study (5%) [49], while 2 studies (9%) employed mixed-methods approaches [26,50].

Preterm infants and/or their parents/caregivers are the focus of the included studies. These studies also evaluated Hospital-to-home transitional care interventions delivered during the NICU stay, at discharge, or shortly after discharge. Across these studies, the interventions include discharge preparation, parental education, home-based follow-up, telehealth, care coordination, and family-centered discharge planning. Individual study characteristics are summarized in Table 4.

### 3.2. Characteristics of Interventions and Outcome Focus

Several hospital-to-home transitional care interventions for the preterm infants and parents were assessed in the studies included in this study. Most of the interventions were multicomponent, where they were initiated during the NICU stay, at discharge, or continued into the post-discharge period [30,32,41,42,45,47]. Structured discharge preparation, parental education, home-based follow-up (home visits or telehealth support), care coordination between hospital and community services, and family-centered care approaches (Family Integrated Care/FiCare) and guided participation were the common components in these interventions [18,19,26,27,29,41,44,45,46,49,50].

The continuity of care was emphasized by several interventions. This involved combining in-hospital preparation with ongoing support after discharge, delivered through telephone follow-up, digital platforms (mobile applications or messaging services), or outpatient follow-up clinics. Some studies also incorporated innovative strategies such as peer support programs, teach-back education, and theory-based discharge readiness models to enhance parental engagement and self-efficacy in infant care [31,44,45,47].

Outcomes were categorized into two groups: infant-related and parent-related outcomes. The infant outcomes are growth parameters (weight gain), hospital readmission, length of hospital stay, breastfeeding outcomes, and healthcare utilization. Parental outcomes primarily focused on caregiving competence, discharge readiness, emotional well-being (stress, anxiety, depression), and satisfaction with care.

Overall, these studies demonstrated a strong emphasis on improving both clinical and psychosocial outcomes through integrated and continuous care approaches. However, the heterogeneity across studies was observed due to the variability in intervention components, delivery methods, outcome measures, and follow-up duration.

### 3.3. Article Quality Assessment Process

**Figure 2:**
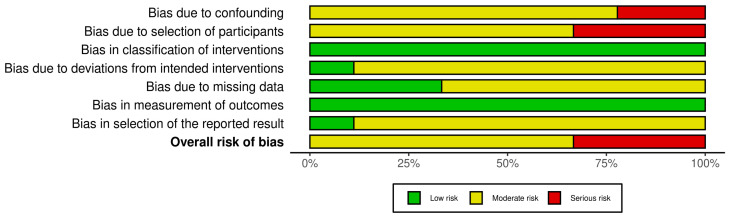
Summary of risk of bias judgments for the included studies using the Risk Of Bias In Non-randomized Studies of Interventions (ROBINS-I) tool.

**Figure 3:**
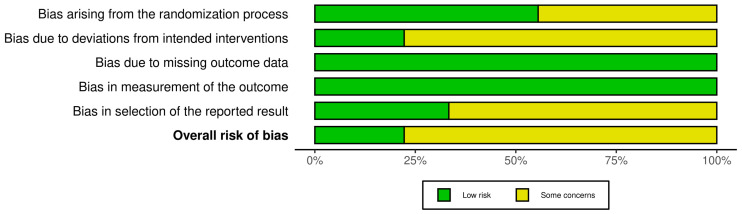
Summary of risk of bias judgments for the included studies using the Risk of Bias 2 (RoB 2) tool.

An overall judgement of risk of bias was assigned for each study based on the criteria of the respective tools. The results of the quality assessment are shown in Figure 2 and Figure 3. Details of the risk-of-bias assessments, including domain-specific judgments for each included study and the corresponding traffic-light plots, are provided in Supplementary File S4.

Risk-of-bias judgments varied according to study design. Among the comparative non-randomized studies assessed using the ROBINS-I tool, six studies were judged to have a moderate overall risk of bias and three studies were judged to have a serious overall risk of bias. The studies rated as having a serious overall risk of bias were Waruingi et al. [40], Toral-López et al. [43], and Mostafanezhad et al. [49]. For Waruingi et al. [40] and Toral-López et al. [43], the serious judgments were mainly related to confounding and selection of participants, including baseline differences between comparison groups and limited control of confounding factors. For Mostafanezhad et al. [49], the serious judgment was primarily related to selection of participants, while the risk of bias due to confounding was judged as moderate. In contrast, the domains related to classification of interventions and measurement of outcomes were generally assessed as low risk across studies.

Meanwhile, the randomized controlled trials were assessed using the RoB 2 tool (Figure 3). In these randomized controlled trials, the overall risk of bias was predominantly rated as low risk or “some concerns”. Most studies demonstrated low risk in the domains of missing outcome data and measurement of outcomes. However, some concerns were identified in the domains related to the randomization process and deviations from intended interventions, largely due to insufficient reporting of allocation of concealment and lack of blinding.

As mentioned previously, studies that had no comparator groups were described in a narrative way. Mainly, these studies reported the feasibility and implementation of transitional care interventions (home-based follow-up, telehealth support, and structured discharge preparation). Comprehensively, the interventions were considered feasible and well accepted by parents. There were also improvements reported in parental confidence, satisfaction, and aspects of infant care such as feeding and growth. Nonetheless, because of the absence of a comparison group, these findings were descriptive in nature. This does not allow conclusions regarding the effectiveness of interventions. Accordingly, these studies were interpreted as providing supportive contextual evidence.

### 3.4. Narrative Synthesis of the Findings

Due to substantial clinical and methodological heterogeneity across the included studies—particularly in intervention components, timing, delivery modes, and outcome measures—a meta-analysis was not feasible. Therefore, following the SWiM guideline, the findings are synthesized by grouping studies based on intervention characteristics and tabulating the direction of effect (e.g., favorable, unfavorable, or no effect) for each outcome domain.

- Impact of Multicomponent and Continuum-of-Care Interventions

The synthesis generally indicated a favorable direction of effect for interventions that adopted a multicomponent, continuum-of-care approach, particularly those initiating preparation before discharge and sustaining follow-up post-discharge. Across studies evaluating multicomponent continuum-of-care models, favorable effects were most commonly observed for infant outcomes, particularly healthcare utilization, feeding, and growth [18,19,26,27,32,41,42]. Although the magnitude of effects varied across studies, interventions that combined discharge preparation with structured post-discharge follow-up generally demonstrated more favorable outcomes than routine care. Several studies reported reductions in emergency department visits or hospital readmissions among infants receiving multicomponent transitional care interventions, although effect estimates and outcome definitions varied across studies. Favorable effects on feeding progression and growth were also reported in several studies; however, differences in outcome measures and follow-up durations limited direct comparison across studies.

In contrast, interventions that relied primarily on a single component—such as in-hospital parental education without structured post-discharge follow-up (e.g., Moradi et al. [30], Mostafanezhad et al. [49])—showed a different pattern. Although these interventions generally reported improvements in parental discharge readiness, favorable effects on infant health outcomes were less consistently observed. Findings related to post-discharge healthcare utilization and infant growth varied across studies, suggesting that improvements in parental knowledge and preparedness alone may not be sufficient to consistently influence infant outcomes in the absence of coordinated post-discharge support.

- Safety and Efficacy of Early Discharge with Home-Based Follow-up

Several studies evaluated early discharge programs supported by home-based nursing care or nasogastric tube feeding follow-up [19,26,27,39,46,50]. These interventions generally reported shorter hospital stays without clear evidence of increased readmissions or adverse events. Several studies also described favorable parental outcomes, including greater confidence and satisfaction with care. Nevertheless, interpretation of these findings should consider differences in study design, sample size, and methodological quality.

- The role of Parental Readiness, Confidence, and Caregiving Competence in Transitional Care

Parent-focused interventions that assessed readiness, confidence, self-efficacy, or caregiving competence included FiCare, teach-back education, guided participation, train-to-home discharge preparation, and theory-based discharge readiness models [29,30,31,32,40,41,42,45,47]. However, these improvements were not consistently linked to better infant outcomes unless they were accompanied by coordinated post-discharge follow-up. The strength and consistency of these associations varied across studies because of differences in intervention characteristics, outcome measures, and study quality.

- Evidence from Non-Comparative Studies

Three studies utilized non-comparative designs [26,49,50], providing contextual evidence regarding feasibility and acceptability rather than effectiveness. All three studies reported that multicomponent, family-centered models and telemedicine-supported discharge programs were feasible to implement and yielded high parental satisfaction. Descriptively, these studies noted reductions in healthcare utilization over time and stable infant growth. However, due to the absence of comparator groups, these favorable directions of effect cannot be solely attributed to the interventions and must be interpreted cautiously.

Table 5 presents a narrative summary of the evidence across outcome domains, organized by comparison group, main outcomes, and overall interpretation of findings. This table was developed to facilitate structured interpretation of the heterogeneous studies included in the review.

### 3.5. Summary of Key Findings

- Transitional care interventions initiated before discharge and continued after discharge appeared to show more favorable patterns across several studies.
- More favorable infant outcomes were reported in several studies evaluating interventions that combined parent education, discharge preparation, and follow-up support. Early discharge with domiciliary nursing or home follow-up was associated with shorter hospital stays in several studies, with no clear evidence of worse infant outcomes.
- Some programs with NICU-to-home or NICU-to-community linkage reported fewer readmissions and lower healthcare utilization.
- Growth, feeding progress, discharge readiness, and transition safety were commonly assessed outcomes, with favorable findings reported in some studies.
- Reduced parental stress, anxiety, and uncertainty may support more responsive caregiving after discharge.
- Telehealth, telephone follow-up, and app-based monitoring may help support continuity of care after NICU discharge, although implementation and outcomes varied across studies.
- Family-centered approaches such as FiCare, COPE, teach-back education, guided participation, and train-to-home models may contribute to improved infant-related outcomes by strengthening parental involvement, but further comparative evidence is needed.
- Findings from non-comparative studies should be interpreted descriptively and cautiously; however, these studies provided useful evidence regarding feasibility and acceptability.

## 4. Discussion

### 4.1. Principal Findings

This review provides a longitudinal perspective on the evolution of hospital-to-home transitional care for preterm infants, showing how interventions have progressed from simple early discharge plans to more family-focused, coordinated, and technology-supported models. A key strength of this review is that it not only summarizes existing evidence but also demonstrates that effective interventions conceptualize discharge as a process rather than a single event.

Interventions appeared to be associated with more favorable outcomes when initiated before discharge, continued after discharge, and addressed both infant monitoring and parental caregiving. Programs that include preparing for discharge, teaching caregivers, and following up are often linked to a safer transition, better feeding support, improved growth, and less healthcare use. The findings suggest that parental outcomes are part of the pathway to infant health because confident, prepared, and capable parents are more likely to recognize feeding problems and complications early.

The pattern of findings also helps explain why some interventions succeeded more than others. Models that combined hospital and community care appeared more likely to be associated with favorable outcomes than interventions limited to education or discharge instructions alone. Interventions that were part of a broader care plan than just isolated counseling seemed more likely to affect infant outcomes because they combined teaching with ongoing support, continuity and access to follow-up. On the other hand, interventions that were not intense, had short follow-up, or had weak links to post-discharge services were less likely to show consistent effects.

The effectiveness of transitional care varies across contexts. In resource-limited settings, priorities often focus on ensuring survival through basic interventions such as breastfeeding support, family education, and home follow-up. In contrast, in resource-rich settings, challenges are more related to care coordination, long-term developmental monitoring, and service integration.

### 4.2. Comparison with Literature

The findings of this review are consistent with the broader neonatal literature suggesting that family-centered and continuity-based interventions may offer broader benefits than discharge preparation alone [51,52]. Studies of kangaroo mother care, family-integrated care, and structured post-discharge support have shown that infant outcomes improve when parents are actively involved, trained, and supported across the transition from hospital to home. This review extends that literature by showing that the same principle applies across multiple types of transitional care, not only in one specific care model [22,28,52,53,54].

The review also supports previous evidence that parental readiness is not simply a psychosocial endpoint but may function as a potential explanatory factor related to infant health after discharge. Several studies in the neonatal literature have shown that parental stress, confidence, and caregiving competence affect feeding, adherence to follow-up, and the early detection of infant problems [11,16,31,55,56,57]. Our review reinforces this relationship by showing that parent-focused interventions are most useful when they strengthen caregiving behaviors that directly influence infant health after discharge.

The review helps explain the variability in intervention effectiveness across studies. Interventions that relied on education alone, without structured follow-up or service linkage, were less consistently effective. This conclusion aligns with previous reviews indicating that educational interventions alone are insufficient to improve infant outcomes unless integrated into a sustained, comprehensive care plan. Interventions requiring complex technological infrastructure or lacking clear interprofessional coordination often faced significant implementation barriers [58,59,60,61]. Interventions relying solely on education, without structured follow-up, yielded inconsistent results.

### 4.3. Implications for Practice

These findings have important implications for healthcare providers involved in neonatal and community care. First, transitional care should be initiated prior to discharge and sustained post-discharge [18,19,26,27,32,41,42,44,45,49,50]. Discharge preparation should not be limited to bedside education; rather, it must be integrated with structured post-discharge follow-up to support caregivers in managing infant care at home [8,12,13,17,18,32,42,45].

Second, effective transitional care requires models that simultaneously monitor infants and support their parents. This can be achieved through home visits, telephone follow-up, telehealth, or app-based monitoring [18,19,26,27,32,46,47]. These approaches are particularly useful for ensuring that parents can feed their infants appropriately, monitor weight gain, recognize warning signs, and seek timely medical care when problems arise [18,19,26,27,32,41,45,46,50]. Such models may be particularly useful when implemented through multidisciplinary collaboration among neonatal clinicians, nurses, physicians, and community health workers [18,44,49].

Third, healthcare systems must move beyond vague support promises and implement structured, clearly defined transitional care packages [8,9,16,18,41,44]. These packages should explicitly outline the responsible providers, initiation timing, intervention duration, information-sharing protocols, and contingency plans for clinical deterioration [18,41,44]. Such care models must balance standardization for safety with the flexibility to adapt to diverse local contexts and individual family needs, ensuring scalability across different healthcare settings.

In Indonesia, this information is crucial because existing maternal and child healthcare systems can be leveraged rather than developing entirely new programs [62]. Current practices, such as the use of the maternal and child handbook, routine infant monitoring, physician referral networks, and community health services, can be integrated to ensure care continuity [63]. A practical approach includes strengthening discharge readiness, providing pre-discharge caregiver education, ensuring structured post-discharge follow-up, monitoring infant growth, supporting breastfeeding, and establishing clear referral pathways. In this way, transitional care can be embedded into the existing healthcare system to ensure timely and continuous care [12].

### 4.4. Study Limitations

This review has several limitations. The included studies were highly heterogeneous in design, intervention components, outcome measures, and follow-up duration, which limited direct comparison and precluded a meta-analysis. Furthermore, several studies utilized non-randomized designs, which restricts their ability to establish causal relationships. A limitation of this review is that only four electronic databases were searched; therefore, relevant studies indexed in other databases such as Embase, CINAHL, Web of Science, and PsycINFO may have been missed, potentially limiting the comprehensiveness of the evidence base. Consequently, studies focusing on nursing, psychosocial, or implementation aspects of transitional care may have been underrepresented.

Additionally, the follow-up periods in several included studies were relatively short, making it difficult to determine whether the initial positive outcomes were sustained over the long term. Methodological limitations were also evident in small sample sizes, single-center designs, or highly specialized settings that may not be representative of general healthcare systems. Finally, studies without comparator groups, while offering valuable descriptive insight regarding feasibility, preclude causal inferences; therefore, their findings must be interpreted with caution compared to robust comparative designs.

The review may also be subject to language bias. Although no language restrictions were applied during the database searches, only English-language full-text publications were included during the screening process because translation resources were unavailable. As a result, potentially relevant studies published in other languages may have been excluded, thereby limiting the comprehensiveness of the evidence base.

Another limitation is that the certainty of evidence was not formally assessed using a framework such as GRADE. Although risk of bias was evaluated at the individual study level, the overall certainty supporting each outcome could not be determined. The substantial heterogeneity in study designs, intervention components, outcome measures, and follow-up durations limited the feasibility of applying a formal certainty-of-evidence assessment. Therefore, the findings should be interpreted with appropriate caution.

Finally, publication and reporting bias were not formally assessed. Studies reporting positive or statistically significant findings may be more likely to be published than studies with null or negative results. Consequently, the overall body of evidence included in this review may overrepresent favorable outcomes associated with hospital-to-home transitional care. In addition, selective reporting of outcomes within individual studies may have influenced the available evidence and should be considered when interpreting the findings.

### 4.5. Future Research

Future research should prioritize multicenter studies to identify what the core, active components of transitional care are and determine the optimal intensity and duration of interventions [22,23,24]. To enable cross-study comparison and future meta-analyses, researchers must adopt standardized, infant-centered outcome measures. Specifically, studies should consistently evaluate weight gain, feeding proficiency, hospital readmission rates, morbidity, and healthcare costs.

More work is also needed to investigate the potential role of parental outcomes in supporting infant health following discharge. Future studies should examine whether improvements in parental confidence, readiness, caregiving competence, and stress levels are associated with better infant outcomes and explore the pathways through which these relationships may occur. Formal mediation analyses may help determine whether parental outcomes function as explanatory factors linking transitional care interventions to infant health outcomes, rather than treating these parental outcomes solely as independent endpoints. Such work would provide a clearer understanding of how transitional care interventions may influence infant outcomes across the post-discharge period [38,51].

Finally, there is a critical need for implementation research in LMICs, such as Indonesia [63]. Future studies should evaluate not only clinical efficacy but also the practical feasibility, cultural acceptability, cost-effectiveness, and scalability of these interventions within existing, resource-constrained healthcare systems [54,63]. Care models that are effective in well-resourced hospitals may not be directly applicable in settings with fewer resources, necessitating context-specific adaptations.

## 5. Conclusions

Hospital-to-home transitional care for preterm infants may offer potential benefits when implemented as a continuous, multicomponent care pathway rather than a single discharge event. Interventions integrating discharge preparation, parental education, and structured post-discharge follow-up were associated with favorable infant outcomes in several studies, including safer transitions home, improved feeding outcomes, and reduced healthcare utilization.

The findings also suggest that parental readiness, confidence, and caregiving competence may play important roles in supporting infant health following discharge. Interventions that enhance parental knowledge and practical caregiving skills may contribute to safer home environments and facilitate the early recognition of potential clinical complications.

Furthermore, the effectiveness of transitional care appears to be context-dependent. In resource-limited settings, simplified family-centered approaches may be beneficial, whereas resource-rich settings may require more comprehensive and integrated models that support long-term developmental follow-up and care coordination. Despite these findings, the available evidence remains heterogeneous, and causal inferences are limited by variability in study designs, intervention components, outcome measures, and risk of bias across studies. Future research should prioritize standardized infant-centered outcomes, clearer reporting of intervention components, and implementation studies evaluating feasibility, scalability, and cost-effectiveness across diverse healthcare settings.

While the findings suggest potential benefits of hospital-to-home transitional care for preterm infants, they should be interpreted with caution. The included studies were heterogeneous in design, intervention components, outcome measures, and follow-up periods, and the overall certainty of evidence could not be formally assessed. In addition, publication and reporting bias may have influenced the available evidence, as studies reporting positive findings are generally more likely to be published than those reporting null or negative results. Selective outcome reporting within individual studies may have also contributed to the predominance of favorable findings observed across the review.

Parental readiness, self-efficacy, and caregiving competence may be important factors associated with improved infant care after discharge, but the included studies do not provide formal evidence of mediation. Coordinated post-discharge follow-up may contribute to improved infant outcomes by supporting parental preparedness; however, the mechanisms underlying these relationships remain uncertain and require further investigation.

## Figures and Tables

**Table 1:** PICOS framework.

Components	Description
**P**	Preterm infants and their parents/caregivers
**I**	Hospital-to-home transitional care models, including discharge preparation, caregiver education, follow-up support, and linkage with community healthcare services
**C**	Usual care includes standard discharge planning, routine NICU care, or no intervention at all
**O**	Infant and parental outcomes related to post-discharge care
**S**	Quantitative and mixed-methods studies, including randomized controlled trials, quasi-experimental, and observational designs

Note: Bold letters indicate the components of the PICOS framework: P, population; I, intervention; C, comparator; O, outcomes; S, study design.

**Table 2:** Search strategy and results.

No	Database	Search Strategy	Results
1	EBSCO-MEDLINE	(“preterm infant *” OR neonate * OR infant OR “low birth weight” OR “extremely premature infant”) AND (“neonatal intensive care unit” OR “intensive care unit *”) AND (“transitional care” OR “transition of care” OR “care transition” OR “discharge planning” OR “hospital to home” OR “post NICU discharge”)	848
2	Scopus	TITLE-ABS-KEY (“preterm infant” OR neonate * OR infant OR “low birth weight” OR “extremely premature infant”) AND TITLE-ABS-KEY (“neonatal intensive care unit” OR “intensive care unit”) AND TITLE-ABS-KEY (“transitional care” OR “transition of care” OR “care transition” OR “discharge planning” OR “hospital to home” OR “post NICU discharge”)	334
3	PubMed	((“preterm infant” OR neonate * OR infant OR “low birth weight” OR “extremely premature infant”) AND (“neonatal intensive care unit” OR “intensive care unit”)) AND (“transitional care” OR “transition of care” OR “care transition” OR “discharge planning” OR “hospital to home” OR “post NICU discharge”)	145
4	Cochrane Library	(“preterm infant” OR neonate * OR infant OR “low birth weight” OR “extremely premature infant”):ti,ab,kw AND (“neonatal intensive care unit” OR “intensive care unit”):ti,ab,kw AND (“transitional care” OR “transition of care” OR “care transition” OR “discharge planning” OR “hospital to home” OR “post NICU discharge”):ti,ab,kw	28

Note: The asterisk (*) indicates a truncation/wildcard symbol used in database searches to retrieve word variants, such as infant and infants, neonate and neonates, or unit and units.

**Table 3:** Inclusion and Exclusion Criteria.

Inclusion Criteria	Exclusion Criteria
**Population:** Preterm infants (born <37 weeks of gestation) and their parents/caregivers	Studies conducted exclusively in-hospital without a transition-to-home component Non-English publications Studies evaluating purely clinical or medical treatments (e.g., pharmacological or procedural interventions) without transitional care components Qualitative-only studies, review articles, editorials, conference abstracts, study protocols, and grey literature
**Setting:** Transition from NICU or hospital to home/community care	
**Intervention:** Structured programs designed to improve continuity of care during the transition from hospital to home. Interventions may include transitional home care, early discharge programs, domiciliary nursing, or Family Integrated Care (FiCare) related to discharge preparation Interventions must include at least one component such as discharge planning with follow-up, home visits, telehealth support, parental education, care coordination, or case management.	
**Comparison:** Usual care, standard discharge care, or no intervention	
**Outcomes:** Infant and/or parental outcomes related to post-discharge care	
**Study designs:** Quantitative and mixed-methods studies were eligible for inclusion. For mixed-methods studies, only the quantitative component contributed to the primary synthesis. Qualitative-only studies were excluded.	
**Time frame:** No restriction on publication year	

Note: Bold text indicates the eligibility criterion categories used to structure the inclusion criteria.

**Table 4:** Characteristics of the included studies.

No.	Author (Year)	Study Design	Sample (Intervention/Control)	Intervention	Comparator	Provider	Follow-Up	Outcome
1	Ortenstrand et al., 1999 [19]	Quasi-experimental study	45/43	Early discharge with domiciliary nursing care; home visits; telephone support; individualized care planning	Routine hospital care	Neonatal nurse; multidisciplinary team	Up to 12 months	LOS, rehospitalization, infections, growth, healthcare use
2	Melnyk et al., 2006 [38]	Randomized controlled trial	137/109	COPE educational-behavioral program delivered in four phases from NICU admission to post-discharge	Routine care	Trained interventionists/nurses	2 months post-discharge	Maternal anxiety, depression, stress, beliefs, mother–infant interaction
3	Sáenz et al., 2009 [39]	Randomized controlled trial	72/68	Early discharge program with PCP follow-up; parental education support; phone calls every 10 days	Standard discharge program	Primary care pediatrician; NICU team; psychologist for assessment	3 months	Parental stress, anxiety, depression, well-being, infant outcomes, healthcare use
4	Waruingi et al., 2014 [40]	Quasi-experimental study	85/85	Special Care Clinic follow-up; discharge planning; growth/development monitoring; care coordination	Historical/standard comparison	Multidisciplinary follow-up clinic team	12 months	ED/urgent care use, growth, follow-up utilization
5	Moyer et al., 2014 [41]	Quasi-experimental study	104/125	Multicomponent NICU-to-ambulatory transition intervention with health coach, enhanced personal health record, and standardized discharge process	Routine discharge care	Health coach; NICU team; PCPs	30 days	Transition quality, caregiver readiness, adverse events, LOS, utilization
6	Brødsgaard et al., 2015 [26]	Mixed-methods study	218	Family-centered early discharge program with parental education, home visits, growth/nutrition monitoring, and community coordination	Not applicable	Specialized neonatal nurse; neonatologist; health visitor; GP	Longitudinal follow-up	Growth, feeding progression, readmission, parental confidence, satisfaction, experiences
7	Ingram et al., 2016 [42]	Quasi-experimental study	Phase 1 (Pre intervention): 128; Phase 2 (Post Intervention): 117	Train-to-Home family-centered discharge planning with train graphic, gestation-specific education, and discharge readiness monitoring	Usual care	Neonatal nurses; multidisciplinary NICU team	8 weeks post-discharge	PMP S-E, LOS, ED visits, outpatient use, costs
8	Toral-López et al., 2017 [43]	Quasi-experimental study	46/40	Early discharge with weekly home follow-up by expert neonatal nurse	Usual care	Expert neonatal nurse; neonatologist	Approximately 2–4 weeks	NOC domains, breastfeeding, LOS, family well-being
9	Liu et al., 2018 [18]	Quasi-experimental study	321/365	Transition Home Plus program with discharge preparation, parental education, care coordination, phone follow-up, home visits, and community linkage	Historical control	Physicians; neonatal nurse practitioners; social workers; family resource specialists	Up to 24 months	Medicaid spending, ED visits, readmissions
10	Moradi et al., 2018 [30]	Randomized controlled trial	30/30	Maternal empowerment program with structured education on infant care, warning signs, bathing, resuscitation, and discharge readiness	Routine care	Nurses; researcher- led education	No post- discharge follow-up	Maternal discharge readiness, LOS
11	Feehan et al., 2019 [44]	Cohort study	549	Multidisciplinary medical home program with continuity from NICU to primary care, developmental surveillance, care coordination, psychosocial screening, and community linkage	Not applicable	PCPs; neonatologists; nurse coordinators; social workers; dietitians; community health workers; referral coordinators; parent advisors	≥2.5 years	Healthcare utilization, psychosocial risk, care coordination, preventive care
12	Van Kampen et al., 2019 [27]	Quasi-experimental study	113/103	Early discharge with nasogastric tube feeding at home; parental training; weekly home visits; continuous support	Standard care	Neonatal and pediatric nurses; neonatologists	3 months	NTF duration, safety, parental satisfaction, growth, breastfeeding, readmission
13	Lee et al., 2019 [45]	Randomized controlled trial	15/15	Guided participation discharge program with three structured education sessions and one follow-up call	Routine care	Advanced neonatal nurse specialist	1 month	PSOC, stress, feasibility, adverse events, retention
14	Fratantoni et al., 2022 [31]	Randomized controlled trial	150/150	Peer support after discharge with parent peer navigators; emotional support; navigation and appointment coordination	Care notebook only/routine care	Trained parent peer navigators	12 months	Stress, depression, anxiety, self-efficacy, infant utilization, development
15	Kaewwimol et al., 2022 [32]	Randomized controlled trial	45/45	Continuity of care program with discharge preparation, readiness assessment, discharge toolkit, home visits, telephone follow-up, and NICU-primary care coordination	Routine care	Nurse researcher; NICU team; primary care providers	4 weeks post-discharge	Parental readiness, performance, service use
16	Zhang et al., 2026 [46]	Randomized controlled trial	100/100	Internet Plus Health Education continuing nursing care; WeChat education; online communication; video training; remote follow-up and monitoring	Routine care	Multidisciplinary health education team; nurses; physician	3 months	Self-efficacy, discharge readiness, satisfaction, infant growth, home environment
17	Tiryaki et al., 2024 [29]	Randomized controlled trial	34/34	Family Integrated Care with structured education, hands-on caregiving training, parental involvement, breastfeeding support, and supervised infant care	Routine care	Neonatal nurses; NICU team; lactation counselor	At discharge	Parental readiness, feeding, breastfeeding, discharge weight, NICU stay
18	Stekelenburg et al., 2024 [47]	Mixed-methods study	26	Baby@Home telemedicine-supported early discharge program with App-based monitoring and weekly telephone consultation	Not applicable	NICU nurses; physician assistants; home care services	2–6 weeks post-discharge	Growth, adverse events, readmission, stress, bonding, feasibility
19	Li et al., 2025 [48]	Randomized controlled trial	37/36	Meleis theory-based discharge readiness linkage program with discharge planning, caregiver training, family ward practice, and WeChat follow-up	Routine care	Multidisciplinary team including neonatologists, nurses, therapists, psychotherapists	1 year	Physical development, readmission, caregiving competence, readiness, anxiety/depression
20	Mostafanezhad et al., 2026 [49]	Quasi-experimental study	33/33	Teach-back-based training with individualized nurse-led sessions, written materials, and repeated verification of understanding	Routine care	NICU nurses	1 month	Maternal discharge readiness, readmission, outpatient visits
21	Segal et al., 2026 [50]	Quasi-experimental study	Pre intervention: 200; Post Intervention: 156	Quality improvement bundle including early discharge planning, standardized 13-item checklists, parental education (resuscitation and basic baby care), and team-based coordination led by a dedicated discharge nurse.	Routine care	Dedicated discharge nurse (coordinating process), neonatologists, and NICU medical/nursing staff	7 days	Primary outcomes: Length of stay (LOS), postmenstrual age (PMA) at discharge, discharge weight, weight percentile at discharge, and readmission rate within 7 days. Secondary outcomes: prematurity-related complications

Note: Sample size refers to the unit of analysis reported in each primary study, which may include preterm infants, parents/caregivers, infant–parent dyads, or pre-/post-intervention cohorts. Therefore, sample sizes are presented according to the reporting structure of the original studies.

**Table 5:** Narrative Summary of Evidence Across Outcome Domains.

Comparison	Main Outcome	Included Studies	Narrative Summary	Overall Interpretation	Methodological Considerations
Multicomponent transitional care vs. routine care	Readmission and healthcare utilization	[18,19,32,42,43,48]	Several studies reported reduced readmission or emergency department visits after structured transitional care interventions	Findings tended to favor transitional care interventions, although heterogeneity remained	Heterogeneity in intervention components and follow-up duration
Early discharge with home follow-up vs. standard discharge	Length of stay and safety	[19,27,46,50]	Early discharge programs were associated with shorter LOS without clear evidence of increased adverse events or readmissions	Generally favorable findings, with methodological variation	Several studies used non-randomized designs
Parent-focused interventions vs. routine care	Parental readiness and confidence	[30,32,40,41,42,47]	Several studies reported improved discharge readiness and caregiving competence	Parent-focused interventions may be beneficial, but findings varied across studies	Outcome measures varied substantially across studies
Telehealth-supported interventions vs. usual care	Continuity of care and follow-up support	[18,41,42,50]	Telehealth interventions may support continuity of care and parental support, although clinical outcomes varied	Potential benefits were observed, but findings remained inconsistent	Small sample sizes and short follow-up periods

## Data Availability

The data supporting the findings of this systematic review are available within the article and its Supplementary Materials. The full extraction dataset is available from the corresponding author upon reasonable request.

## Supplementary Materials

The following supporting information can be downloaded at: https://www.mdpi.com/article/10.3390/children13070876/s1, Supplementary File S1: PRISMA 2020 checklist; Supplementary File S2: Search strategies; Supplementary File S3: Data extraction sheet; Supplementary File S4: Risk-of-bias assessments and traffic-light plots; Supplementary File S5: List of excluded full-text studies and reasons for exclusion.

## Abbreviations

The following abbreviations are used in this manuscript:

**BPD**	Bronchopulmonary dysplasia
**COPE**	Creating Opportunities for Parent Empowerment
**ED**	Emergency Department
**FiCare**	Family Integrated Care
**GA**	Gestational age
**HAMA**	Hamilton Anxiety Scale
**HAMD**	Hamilton Depression Scale
**HRU**	Healthcare resource use
**ICU**	Intensive care unit
**LMICs**	Low- and middle-income countries
**LOS**	Length of stay
**NICU**	Neonatal intensive care unit
**NOC**	Nursing Outcomes Classification
**NTF**	Nasogastric tube feeding
**PMA**	Postmenstrual age
**PICOS**	Population, Intervention, Comparison, Outcome, Study design
**PCP**	Primary care pediatrician
**PMP S-E**	Perceived Maternal Parenting Self-Efficacy
**PRISMA**	Preferred Reporting Items for Systematic Reviews and Meta-Analyses
**PROSPERO**	International Prospective Register of Systematic Reviews
**PSOC**	Parenting Sense of Competence
**RHDS**	Readiness of Parents for Discharge Scale
**RCTs**	Randomized controlled trials
**RoB 2**	Cochrane Risk of Bias 2
**ROBINS-I**	Risk Of Bias In Non-randomized Studies of Interventions
**SWiM**	Synthesis Without Meta-analysis
**WHO**	World Health Organization
