# Supplementary material for: The Effect of Transition to Home Care Model on the Outcomes of Premature Infants and Their Parents: A Systematic Review
Source: Children (Basel). 2026 Jun 30;13(7):876. doi: 10.3390/children13070876 (PMC13407022; doi:10.3390/children13070876)
Supplement: Supplementary file 1 [file children-13-00876-s001.zip › Supplementary File S2_Search Strategies.pdf]

Supplementary File 2. Search Strategies

Literature Search Strategy
The initial database search was conducted in March 2025 and updated in March 2026 to identify newly published studies eligible for inclusion in the review.
The searches were performed in the following electronic databases:
1. EBSCO-MEDLINE
2. Scopus
3. PubMed
4. Cochrane Library

The search strategy was developed with assistance from an experienced medical librarian and used database-specific syntax and free-text terms related to preterm infants, neonatal intensive care units, and hospital-to-home transitional care. No publication-year restrictions were applied. English-language eligibility was applied during the screening process rather than as a database search filter because resources for translation of non-English articles were not available. Grey literature was not searched. Reference lists of included studies were screened manually to identify additional eligible studies. No separate controlled vocabulary terms, such as MeSH terms, were manually combined with the free-text strategy. In PubMed, Automatic Term Mapping was allowed where applicable. The full electronic search strategies used for each database are presented below.

No	Database	Search Date	Exact search string as entered	Field tags / database-specific syntax	Filters or limits applied	Records retrieved
1	EBSCO-MEDLINE	Initial search: March 2025; updated: March 2026	("preterm infant*" OR neonate* OR infant OR "low birth weight" OR "extremely premature infant") AND ("neonatal intensive care unit" OR "intensive care unit*") AND ("transitional care" OR "transition of care" OR "care transition" OR "discharge planning" OR "hospital to home" OR "post NICU discharge")	The search was entered as a free-text Boolean search in EBSCO-MEDLINE. No additional manual subject heading or field-tag restriction was applied.	No publication-year filter was applied. English-language eligibility was applied during screening rather than as a database search filter.	848
2	Scopus	Initial search: March 2025; updated: March 2026	TITLE-ABS-KEY ("preterm infant" OR neonate* OR infant OR "low birth weight" OR "extremely premature infant") AND TITLE-ABS-KEY ("neonatal intensive care unit" OR "intensive care unit") AND TITLE-ABS-KEY ("transitional care" OR "transition of care" OR "care transition" OR "discharge planning" OR "hospital to home" OR "post NICU discharge")	The Scopus field tag TITLE-ABS-KEY was used to search within titles, abstracts, and keywords.	No publication-year filter was applied. English-language eligibility was applied during screening rather than as a database search filter.	334
3	PubMed	Initial search: March 2025; updated: March 2026	(("preterm infant" OR neonate* OR infant OR "low birth weight" OR "extremely premature infant") AND ("neonatal intensive care	The search was entered using PubMed free-text Boolean syntax. No manual field tags were	No publication-year filter was applied. English-language eligibility was applied during screening	145

No	Database	Search Date	Exact search string as entered	Field tags / database-specific syntax	Filters or limits applied	Records retrieved
			unit" OR "intensive care unit")) AND ("transitional care" OR "transition of care" OR "care transition" OR "discharge planning" OR "hospital to home" OR "post NICU discharge")	applied. No separate MeSH terms were manually added. PubMed Automatic Term Mapping was allowed where applicable.	rather than as a database search filter.	
4	Cochrane Library	Initial search: March 2025; updated: March 2026	("preterm infant" OR neonate* OR infant OR "low birth weight" OR "extremely premature infant"):ti,ab,kw AND ("neonatal intensive care unit" OR "intensive care unit"):ti,ab,kw AND ("transitional care" OR "transition of care" OR "care transition" OR "discharge planning" OR "hospital to home" OR "post NICU discharge"):ti,ab,kw	The Cochrane Library field tag :ti,ab,kw was used to search within titles, abstracts, and keywords.	No publication-year filter was applied. English-language eligibility was applied during screening rather than as a database search filter.	28

Abbreviations: EBSCO-MEDLINE, MEDLINE via EBSCOhost; MeSH, Medical Subject Headings; NICU, neonatal intensive care unit; TITLE-ABS-KEY, title, abstract, and keyword search fields in Scopus; ti,ab,kw, title, abstract, and keyword fields in the Cochrane Library.
