# Supplementary material for: The Effect of Transition to Home Care Model on the Outcomes of Premature Infants and Their Parents: A Systematic Review
Source: Children (Basel). 2026 Jun 30;13(7):876. doi: 10.3390/children13070876 (PMC13407022; doi:10.3390/children13070876)
Supplement: Supplementary file 1 [file children-13-00876-s001.zip › Supplementary File S4_Risk of Bias Assessment and Traffic-Light Plots.pdf]

Supplementary File 4: Risk of Bias Assessments

Table S4a. Risk of bias judgments for the included studies using the Cochrane Risk of Bias 2 (RoB 2) tool

Study	Bias arising from the randomization process	Bias due to deviations from intended interventions	Bias due to missing outcome data	Bias in measurement of the outcome	Bias in selection of the reported result	Overall
Melnyk 2006	Low	Low	Low	Low	Low	Low
Saenz 2009	Low	Some concerns	Low	Low	Some concerns	Some concerns
Moradi 2018	Some concerns	Some concerns	Low	Low	Some concerns	Some concerns
Lee 2019	Low	Some concerns	Low	Low	Low	Some concerns
Fratantoni 2022	Low	Low	Low	Low	Low	Low
Kaewwimol 2022	Some concerns	Some concerns	Low	Low	Some concerns	Some concerns
Zhang 2026	Some concerns	Some concerns	Low	Low	Some concerns	Some concerns
Tiryaki 2024	Low	Some concerns	Low	Low	Some concerns	Some concerns
Li 2025	Some concerns	Some concerns	Low	Low	Some concerns	Some concerns

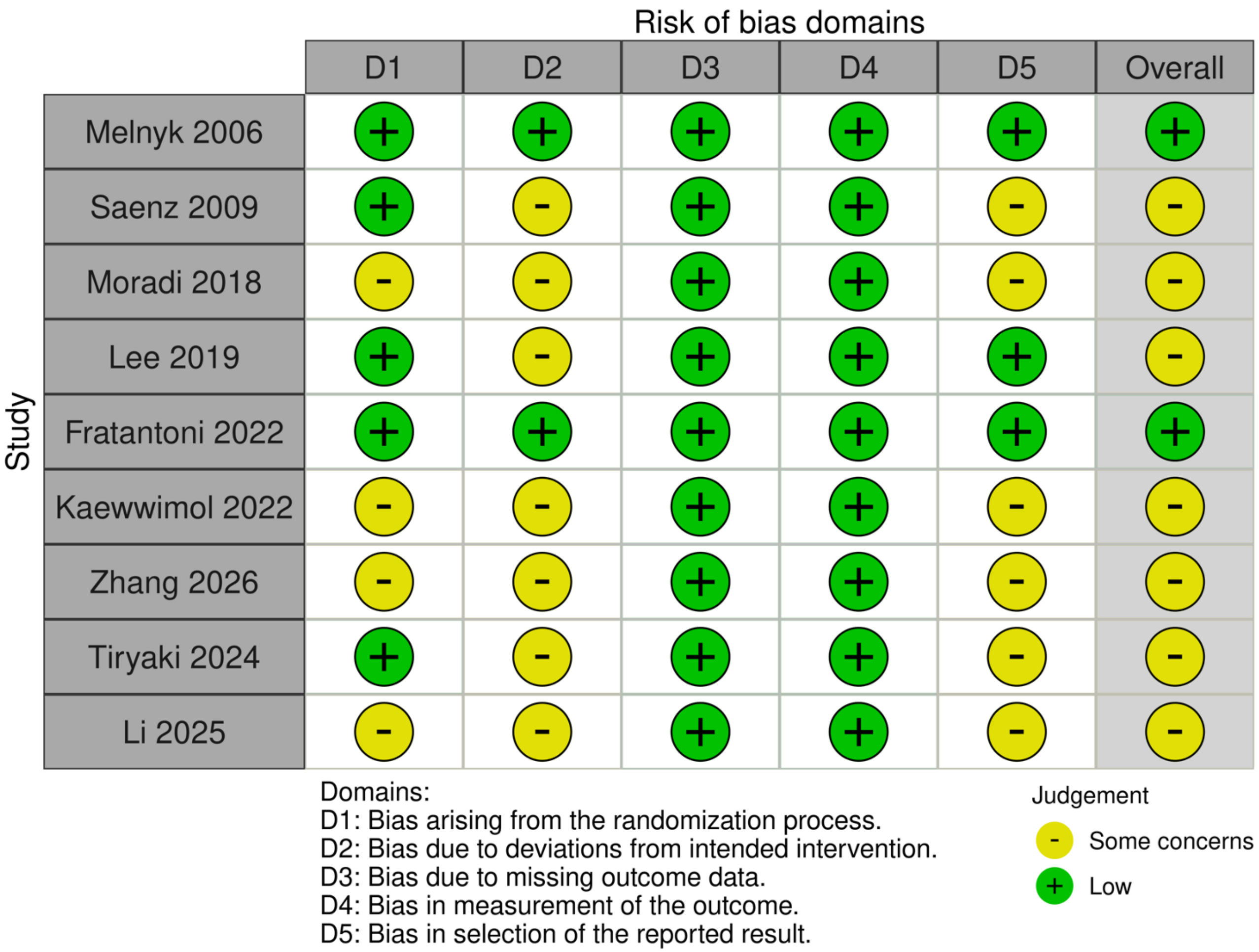

Figure S4a : Risk of bias judgments for the included studies using the Cochrane Risk of Bias 2 (RoB 2) tool

Table S4b. Risk of bias judgments for the included studies using the Risk Of Bias In Non-randomized Studies of Interventions (ROBINS-I) tool

Study	Bias due to confounding	Bias in selection of participants	Bias in classification of interventions	Bias due to deviations from intended interventions	Bias due to missing data	Bias in measurement of outcomes	Bias in selection of the reported result	Overall
Ortenstrand 1999	Moderate	Moderate	Low	Moderate	Moderate	Low	Moderate	Moderate
Waruingi 2014	Serious	Serious	Low	Moderate	Moderate	Low	Moderate	Serious
Moyer 2014	Moderate	Moderate	Low	Low	Low	Low	Low	Moderate
Ingram 2016	Moderate	Moderate	Low	Moderate	Moderate	Low	Moderate	Moderate
Toral-López 2017	Serious	Serious	Low	Moderate	Moderate	Low	Moderate	Serious
Liu 2018	Moderate	Moderate	Low	Moderate	Low	Low	Moderate	Moderate
van Kampen 2019	Moderate	Moderate	Low	Moderate	Moderate	Low	Moderate	Moderate
Mostafanezhad 2026	Moderate	Serious	Low	Moderate	Moderate	Low	Moderate	Serious
Segal 2026	Moderate	Moderate	Low	Moderate	Low	Low	Moderate	Moderate

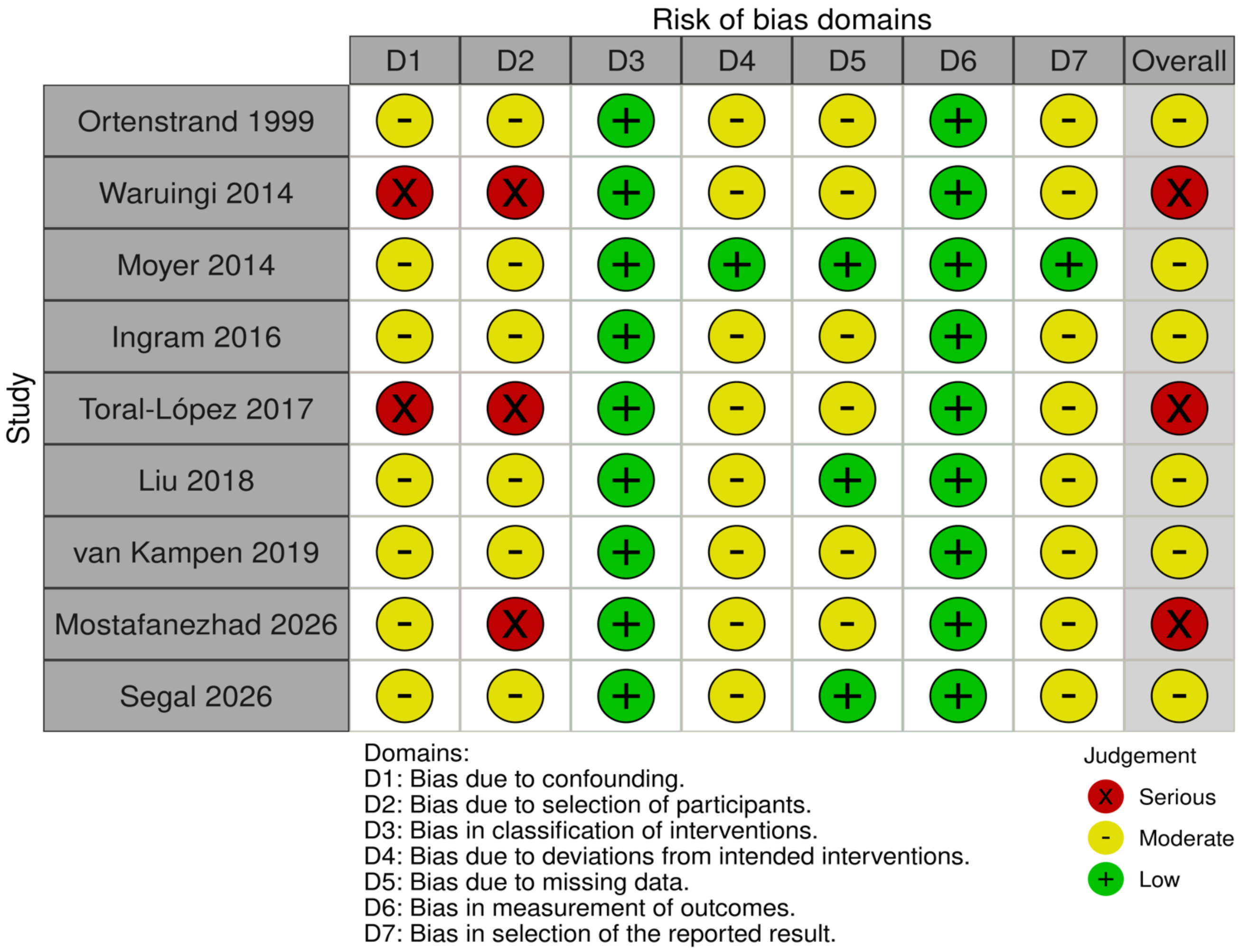

Figure S4b : Risk of bias judgments for the included studies using the Risk Of Bias In Non-randomized Studies of Interventions (ROBINS-I) tool
